# Positive Clinical Signs of Functional Neurological Disorders Are Found in Patients With Functional Cognitive Disorders

**DOI:** 10.1002/brb3.71640

**Published:** 2026-08-19

**Authors:** Olga Goyena, Paulina Soto, Charlotte Joly, Juliette Palisson, Angeline Maillard, Inas Al Chare, Kenza Benrahmoune, Elsa Mhanna, Beatrice Garcin

**Affiliations:** ^1^ Functional Neurological Disorder Unit, FHU Treat FND Sorbonne Paris Nord University, Avicenne Hospital, APHP Bobigny France; ^2^ Frontlab Team Brain Institute, INSERM U1127, Salpêtrière Hospital Paris France

## Abstract

**Introduction:**

The proposition of diagnostic criteria of functional cognitive impairment (FCD) is relatively recent, includes different subtypes of heterogenous aetiologies, and likely involves underlying mechanisms similar to those found in functional neurological disorders (FNDs). The present study aims at describing clinical features in a group of patients with FCD in order to identify positive signs of FCD and the overlap with FND.

**Materials and Methods:**

We prospectively included patients with FCD and patients with FND with disabling cognitive complaints seen in the Neurology department of Avicenne Hospital (Bobigny, France), which houses a memory clinic and a multidisciplinary FND unit.

**Results:**

We included 39 patients between April 2023 and December 2024, 19 having isolated FCD, and 20 FND with cognitive features (FND + FCD). In the systematic symptom checklist, memory impairment was the most frequent cognitive complaint, reported by all patients. Among patients classified as having FND + FCD, 95% presented at least one positive sign of FND on neurological examination, while 42.1% of those classified as having FCD alone showed at least one of the positive signs.

**Conclusion:**

This study highlights the potential utility of screening for positive clinical signs in patients presenting with FCD and the overlap between FND and FCD.

## Graphical abstract

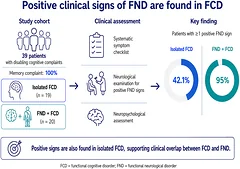

## 1 Introduction

Functional cognitive disorders (FCDs) are characterized by persistent and distressing subjective cognitive complaint (Pennington et al. 2019) with evidence of internal inconsistency that cannot be better explained by another medical or psychiatric condition (McWhirter et al. 2022). They can lead to functional impairment and have a significant impact on daily life. They are frequently associated with diagnosis delays and generate considerable costs, both in terms of healthcare utilization and social consequences, including work cessation and claims for unemployment or disability benefits, which are commonly reported in FCD (Cheval et al. 2024).

The proposition of diagnostic criteria for FCDs is relatively recent and encompasses a spectrum of clinical presentation and severity. FCDs are reported in a significant proportion of individuals attending memory clinics (Ball et al. 2021), with prevalence particularly high among older adults. In the LIFE study, conducted in a German population, 53% of adults aged 40–79 reported subjective memory complaints (Luck et al. 2018). Similarly, a U.S. population‐based survey found that 11.1% of adults over 45 years of age reported concerns about their cognition (Luck et al. 2018).

FCDs have heterogeneous aetiology and likely involve underlying mechanisms (Pennington et al. 2019), possibly paralleling those involved in functional neurological disorders (FNDs). The two conditions share predisposing and perpetuating risk factors as well as precipitating events. These overlaps have fuelled debate over whether they are distinct clinical entities or fall in the same continuum.

Comorbidities, whether acting as precipitating factors (including neurological conditions) or as co‐occurring functional somatic syndromes, are frequently reported in FCD. Cognitive symptoms are also described in people with functional somatic disorders (e.g., irritable bowel syndrome, chronic fatigue syndrome, and fibromyalgia) (Teodoro et al. 2018), with impairment of executive function and selective attention. This overlap may further complicate the differential diagnosis.

Diagnostic confirmation in FCD remains challenging owing to symptom evolution over time, the frequent co‐occurrence of comorbidities (Ball et al. 2021), and limited availability or delayed access to investigations that help exclude neurodegenerative disease, such as cerebrospinal fluid biomarkers and advanced neuroimaging, including nuclear medicine techniques. As a result, the diagnostic process is often complex with diagnostic uncertainty, which has important implications for both clinical care and research. Consequently, detailed reporting of demographic and clinical characteristics is essential.

Given these challenges, better diagnostic tools for FCD are needed, beginning with robust clinical phenotyping to identify diagnostically useful signs and patterns. One such pattern is symptom inconsistency and variability: patients are often multisymptomatic yet do not meet criteria for neurodegenerative disease, have normal paraclinical investigations, experience abrupt fluctuations over short intervals, and maintain coherent, well‐organized speech. In a Scottish study aimed at improving the accuracy and safety of FCD diagnosis, the most predictive feature was the length and level of detail in responses to an open‐ended question (McWhirter et al. 2022). Despite this, diagnostic uncertainty remains common, leaving many patients in a prolonged diagnostic limbo characterized by repeated referrals, inconclusive investigations, and unclear treatment pathways (Ball et al. 2021).

The identification of positive clinical signs in FND has improved patient care and catalyzed research into pathophysiology and treatments. FNDs are no longer diagnoses of exclusion; they can now be identified with precision and thus studied and treated (McWhirter et al. 2022). Similarly, we propose that improving the diagnosis of FCD hinges on defining positive clinical features (Ball et al. 2021), including findings from neurological examination and neuropsychological testing. Such assessments can yield clinically useful markers, including characteristic cognitive profiles. Moreover, recognizing positive clinical signs commonly associated with FND in patients presenting with FCD would both strengthen a positive FCD diagnosis and support the view that these conditions are manifestations of a possible single clinical entity.

This study aims at describing clinical features of FCD that include both patients presenting with isolated cognitive complaints and those with comorbid FND, integrating highly specific FND clinical signs.

## 2 Materials and Methods

### 2.1 Study Design, Inclusion, and Exclusion Criteria

This is a prospective observational study conducted at the Neurology Department of Avicenne Hospital (Bobigny, France), which hosts a memory clinic and a multidisciplinary FNDs unit.

Adult patients seen between April 2023 and December 2024 with diagnoses consistent with FCD or FND with significant cognitive complaints fulfilling diagnostic criteria of FCD (Ball et al. 2020) were eligible for inclusion, provided they met the following criteria: age 18–80 years, native French speaker, and educational level of primary school certificate (Certificat d’études primaires, CEP) or higher. In the FND + FCD group, cognitive complaints were considered clinically significant if they were listed among the patient's three most disabling symptoms. Exclusion criteria included neurological comorbidities, such as multiple sclerosis or Parkinson's disease; unstable psychiatric comorbidity; other significant medical comorbidities, such as cancer requiring active treatment; clinical features suggestive of a neurodegenerative disorder, including visual hallucinations, REM sleep behavior disorder, frontal syndrome, or non‐functional parkinsonism; and abnormalities on ancillary investigations, including brain MRI, CSF analysis, or 18F‐FDG PET when performed. For vascular disease, patients were excluded in the presence of a vascular lesion larger than 2 cm or vascular leukoencephalopathy with a Fazekas score greater than 2.

### 2.2 Patient Selection

Patients seen in the memory clinic for cognitive complaints were included in the FCD group, and patients attending the FND unit for sensorimotor symptoms were included in the FND + FCD group.

Patients were selected based on the diagnostic criteria for “Functional cognitive disorder" (Ball et al. 2020) for the FCD group and based on DSM‐5 criteria for FND for the FND + FCD group.

Other causes of cognitive impairment were excluded by a standardized work‐up comprising blood tests (including vitamin B12 and infectious serologies), brain MRI in all participants, and electroencephalography when indicated. Abnormal MRI and/or vascular disease (Fazekas score > or = 2) were criteria for exclusion. Additionally, 41% of all patients underwent either lumbar puncture with CSF Alzheimer's disease biomarkers or 18F‐FDG PET when clinically indicated to verify normality. All FDG‐PET scans and lumbar punctures performed in the cohort were unremarkable, with no metabolic pattern suggestive of neurodegenerative disease or another neurological condition.

### 2.3 Data Collection

Patients were recruited during neurological consultation at the memory clinic or at the FND unit. A comprehensive evaluation was conducted in a day clinic setting, including both neurological and neuropsychological assessments. Clinical data were recorded using a standardized assessment form completed by the neurologist, and electronic medical records were reviewed to ensure data accuracy and completeness.

The collected variables included age of symptom onset and age at the time of neuropsychological testing, gender, educational level, alcohol and tobacco consumption, neurological, psychiatric, functional, immune‐mediated, and other relevant medical history. Additionally, complementary tests performed, employment status, identified precipitating factors, detailed cognitive complaints, and additional reported symptoms based on systematic checklists filled out by the patient, and findings from the neurological examination were recorded, with particular attention to positive clinical signs characteristic of FNDs. Patients were examined by a neurologist of the department, experienced in FND, and sign positivity was defined as previously described in the literature (detailed checklist in Table 5 and the Supporting Information) (Aybek and Perez 2022) (Daum et al. 2014).

A comprehensive neuropsychological assessment was conducted by a neuropsychologist using multiple standardized batteries and questionnaires, with overlapping measures across cognitive domains. This approach was specifically designed to characterize patients’ cognitive profiles. The following cognitive domains were covered:

- *Global cognition*: Mini‐Mental State Examination (MMSE).
- *Executive functioning*: Verbal and visual working memory tasks, Batterie Rapide d'Efficience Frontale (BREF), Trail Making Test, Stroop Test, SimiCAT, verbal phonemic fluency.
- *Episodic memory*: FCSRT; Rey‐Osterrieth Complex Figure (delayed recall); DMS‐48.
- *Language*: 40‐item Naming Test (DO‐40), verbal semantic fluency.
- *Visual attention*: Bells Cancellation Test.
- *Praxis/visuoconstruction*: Batterie des Praxies de Mahieux, Rey–Osterrieth Complex Figure (copy), Figures de la BEC‐96.
- *Validity test*: Rey 15‐Item Test.

For each neuropsychological test, performance was classified as impaired or preserved according to validated normative data and established cut‐off scores specific to that test.

Detailed descriptions of all tests are presented in in supplementary table 4.

### 2.4 Statistical Analysis

Statistical analyses were performed using Microsoft Excel software. Categorical variables are presented as counts (percentages), and continuous variables as mean (standard deviation) or median (interquartile range), according to distribution. Between‐group comparisons used Student's *t* test for continuous variables, a chi‐square test for categorical variables, and Fisher's exact test for qualitative variables.

Given the exploratory nature of the study and the limited sample size, the primary interpretation of between‐group comparisons was based on uncorrected exact *p*‐values. However, Bonferroni‐corrected *p*‐values were also reported in the tables for transparency and to allow readers to assess the robustness of the findings.

### 2.5 Ethics

All participants provided oral consent after written and oral information, as required by French legislation for research that does not significantly alter usual care. The study protocol was approved by the Ethics Committee “Comité Local Ethique d'Avicenne” (approval registered under number CLEA‐2024‐no. 367).

## 3 Results

### 3.1 Demographic Characteristics

A total of 41 patients were identified, of whom two were excluded: one due to severe vitamin B12 deficiency and another due to uncertainty about a possible underlying cerebellar degenerative process, as noted on neurological examination.

Of the 39 patients evaluated, 19 were classified as having an FCD, having consulted in the Neurology Department exclusively for cognitive complaints, although additional functional signs or symptoms may have been identified during history taking and examination. The remaining 20 patients were classified as having FND with cognitive complaints (FND), having been referred to the FNDs Unit for multiple neurological symptoms, including cognitive complaints.

Most patients were women (82.1%), with a mean age at symptom onset of 43.7 years (SD: 13.2). Patients with FCD had a mean age at onset of 50.26 years (SD: 10.4), whereas the FND group had a significantly earlier age of onset, with a mean age of 37.7 years (SD: 12.9). The overall mean age at the time of neuropsychological testing was 51.6 years (SD: 10.3), with no statistically significant differences between the two groups. There were also no statistically significant differences in other demographic variables, such as alcohol consumption (28.2%), tobacco use (33.3%), or the use of psychotropic medications (48.7%). (Other demographic characteristics summarized in Table 1).

**TABLE 1:** Demographic characteristics.

	FCD (*n* = 19)	FND (*n* = 20)	Total (*n* = 39)	*p* VALUE	BONFERONNI CORRECTED *p* VALUE
**Women**	15 (78.9%)	17 (85%)	32 (82.1%)	0.695	1
**Age at symptoms onset**	50.3 (10,4)	37.7 (12.9)	43.7 (13.2)	0.00197*	0.0171*
**Age at assessment**	54.4 (9.6)	49.2 (10.6)	51.6 (10.3)	0.120	1
**Years of schooling**	13.47 (3.6)	10.46 (3.8)	11.40 (3.7)	0.0169*	0.1395
**Alcohol consumption**	6 (31.5)	5 (2)	11 (28.2)	0.731	1
**Smoking**	6 (31.5)	7 (3)	13 (33.3)	1	1
**% Who had either LP of FDG‐TEP**	8 (42.1)	8 (4)	16 (41.0)	1	1
**% Receiving psychotropic medication**	11 (57.8)	8 (4)	19 (48.7)	0.343	1
**% Occupational inactivity due to FND/FCD**	6 (31.5)	14 (7)	20 51.2)	0.0256*	0.2304

Abbreviations: FDG‐TEP: [^18^F]‐fluorodeoxyglucose positron emission tomography; LP: lumbar puncture.

^*^ *p* < 0.05.

Regarding years of schooling, the FCD group had significantly more years of schooling than the FND group, with a mean of 13.47 years compared with 10.46 years, respectively; as for occupational disability, 51.3% of patients were on medical leave due to neurological symptoms, with significant differences in proportion between both groups: 14 patients in the FND group (70% of that group) and only six patients in the FCD group (31.6% of that group).

All patients had at least one comorbidity, most commonly classified as “other,” including diabetes, hypertension, overweight, and prior surgery. Overall, 41% had a neurological history, most frequently headaches. Additionally, 25.6% of patients had a diagnosis of another functional somatic disorder, with fibromyalgia being the most prevalent. Psychiatric history was also common (69.2%), particularly depression (61.5%), followed by anxiety (43.6%), while only one patient in the entire group had a history of psychotic disorders. More than half of patients (56.4%) also reported physical or psychological trauma during childhood.

Most patients (76.9%) identified a precipitating factor. The most common precipitating factor was COVID‐19 infection. Emotional trauma tended to be more frequent in the FND group, while burnout was more common in the FCD group. No significant between‐group differences were observed in the frequency or type of precipitating factors, either before or after Bonferroni correction for multiple comparisons (see Table 2).

**TABLE 2:** Precipitating factors.

	FCD (*n* = 19)	FND (*n* = 20)	Total (*n* = 39)	*p* value	Bonferroni corrected *p* value
**Precipitating Factor *n* (%)**	**14 (73.68)**	**17 (85)**	**30 (76.92)**	0.451	1
Head trauma *n* (%)	3 (21.4)	4 (23.5)	7 (23.3)	1	1
Infection (COVID) *n* (%)	4 (28.5)	6 (35.2)	10 (33.3)	1	1
Emotional trauma *n* (%)	1 (7.1)	5 (29.4)	6 (2)	0.185	1
Burn‐out *n* (%)	4 (28.5)	2 (11.7)	6 (2)	0.370	1
Others *n* (%)	2 (14.2)	0	2 (5.1)	0.196	1

### 3.2 Cognitive Complaints and Other Reported Symptoms

In the systematic symptom checklist, memory impairment was the most frequent cognitive complaint, reported by all patients, followed by attention deficit (87%), brain fog (64%), and language impairment (56%). Significant differences were observed only for brain fog before correction for multiple comparison, which was more frequent in the FND group. The distribution of cognitive complaints is shown in Table 3.

**TABLE 3:** Cognitive complaints.

	FCD (*n* = 19)	FND (*n* = 20)	Total (*n* = 39)	*p* value	Bonferroni corrected *p* value
**Memory**	19 (100)	20 (100)	39 (100)	1	1
**Attention**	17 (89.4)	17 (85)	34 (87.1)	1	1
**Brain fog**	9 (47.3)	16 (80)	25 (64.1)	0.048*	0.484
**Language**	10 (52.6)	12 (60)	22 (56.4)	0.751	1
**Organization**	10 (42.6)	11 (55)	21 (53.8)	1	1
**Orientation**	5 (26.3)	10 (50)	15 (38.4)	0.191	1
**Visual disturbances**	3 (15.7)	6 (30)	9 (23.)	0.451	1
**Praxis**	2 (10.5)	0	2 (5.1)	0.231	1
**Behaviour problems**	3 (15.7)	4 (20)	7 (17.9)	1	1
**Recognition of people**	2 (10.5)	3 (15)	5 (12.8)	1	1

^*^ *p* < 0.05.

In addition to cognitive complaints, several patients reported other symptoms during the clinical systematic interview (Table 4). Fatigue and pain were the most frequently reported symptoms across the entire patient group, with significant differences between the two groups. In the FND group, fatigue and pain were reported by 95% and 80% of patients, respectively, while these symptoms were less prevalent in the FCD group, although still notable, at 68% and 47%. Furthermore, tremor, motor deficits, sensory disturbances, and urinary symptoms were significantly more frequent in the FND group compared to the FCD group. After Bonferroni correction for multiple comparisons, tremor, motor deficit, and urinary symptoms remained significantly more frequent in the FND group than in the FCD group.

**TABLE 4:** Other reported symptoms.

	FCD (*n* = 19)	FND (*n* = 20)	Total (*n* = 39)	*p* value	Bonferroni corrected *p* value
**Dissociative seizures**	2 (10.5)	6 (30)	8 (20.5)	0.235	1
**Dystonia**	0	3 (15)	3 (7.6)	0.231	1
**Tremor**	1 (5.2)	12 (60)	13 (33.3)	0.0004*	0.00518*
**Motor deficit**	3 (15.7)	13 (65)	16 (41.0)	0.003*	0.0367*
**Sensory symptoms**	3 (15.7)	12 (60)	15 (38.4)	0.007*	0.0949
**Pain**	9 (47.3)	16 (80)	25 (64.1)	0.048*	0.580
**Fatigue**	13 (68.4)	19 (95)	32 (82.0)	0.043*	0.523
**Speech or phonation problems**	3 (15.7)	8 (40)	11 (28.2)	0.155	1
**Dizziness**	6 (31.5)	12 (60)	18 (46.1)	0.111	1
**Dysphagia**	1 (5.2)	2 (10)	3 (7.6)	1	1
**Urinary symptoms**	1 (5.2)	11 (55)	12 (30.7)	0.0012*	0.015*
**Digestive symptoms**	6 (31.5)	9 (45)	15 (38.4)	0.514	1

^*^ *p* < 0.05.

### 3.3 Neurological Examination

Among patients classified as FND, 95% exhibited at least one positive sign on examination, whereas 42.11% of those classified as FCD showed at least one positive sign, with a mean of four signs per patient. In the FCD group, the most frequent signs were excessive slowness without bradykinesia, hypoesthesia excessively respecting the midline, Hoover's sign, the abductor sign, and oculomotor discomfort. Positive FND signs identified on neurological examination are presented in Table 5.

**TABLE 5:** Positive clinical signs of functional neurological disorder (FND).

	FCD (*n* = 19)	FND (*n* = 20)	Total (*n* = 39)	*p* value
Number of patients showing at least one positive FND sign, *n* (%) Mean number of positive signs in the group	8 (42.11) 3.1	19 (95) 5.3	27 (69.23)	
**Walking**	*n* (%)	*n* (%)	*n* (%)	
Sudden knee buckling	1 (12.5)	7 (36.8)	8 (29.6)	0.044
Dragging monoplegic leg	0	5 (26.3)	5 (18.5)	0.047
Non‐economic posture	0	6 (31.5)	6 (22.2)	0.020
Hesitant/cautious gait	3 (37.5)	10 (52.6)	13 (48.1)	0.054
**Motor**	*n* (%)	*n* (%)	*n* (%)	
Excessive slowness without bradykinesia	4 (50)	3 (15.7)	7 (25.9)	0.695
Collapsing/give‐away weakness	3 (37.5)	16 (84.2)	19 (7.3)	<0.001
Drift without pronation	1 (12.5)	6 (31.5)	7 (25.9)	0.091
Hoover's sign	3 (37.5)	13 (68.4)	16 (59.2)	0.005
Abductor sign	3 (37.5)	9 (47.3)	12 (44.4)	0.103
**Sensory**	*n* (%)	*n* (%)	*n* (%)	
Midline splitting	3 (37.5)	6 (31.5)	9 (33.3)	0.451
Splitting of vibration	1 (12.5)	6 (31.5)	7 (25.9)	0.091
**Others**	*n* (%)	*n* (%)	*n* (%)	
Discordance	2 (25)	2 (10.5)	4 (14.8)	1
Distractibility	1 (12.5)	7 (35)	8 (29.6)	0.044
Entrainment effect	0	3 (15.7)	3 (11.1)	0.231
Convergence spasm or oculomotor discomfort	0	1 (5.2)	1 (3.7)	1

*Note*: Checklist of positive clinical signs of FND collected during examination (Daum et al. 2014; Aybek and Perez 2022) (for more information on how to perform this clinical examination, see Table 3. Results are presented as number of patients (% of patients).

^*^ *p* < 0.05.

### 3.4 Neuropsychological Evaluation

**FIGURE 1:**
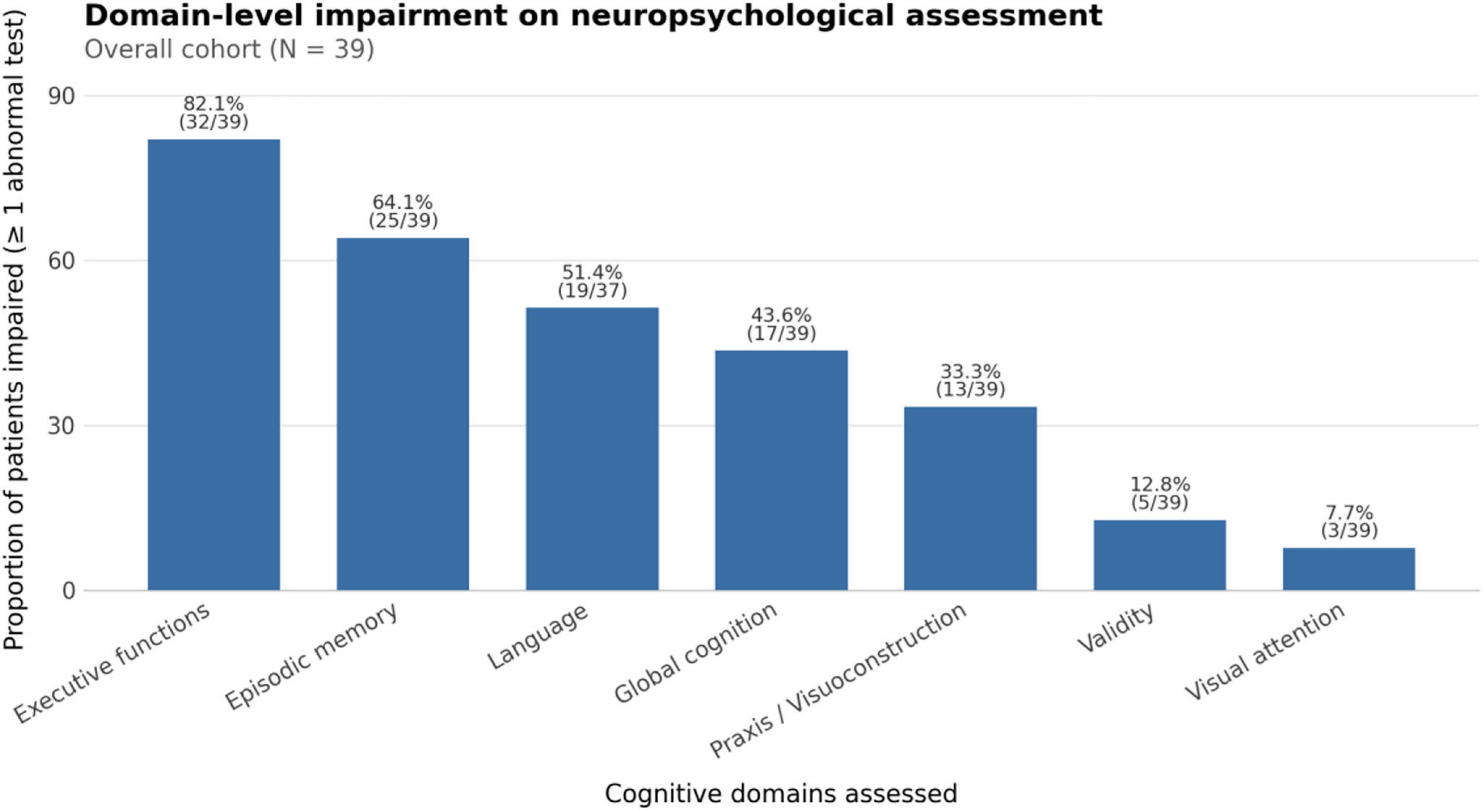
Domain‐level impairment on neuropsychological assessment.

Most patients showed impairment in at least one cognitive domain. Executive function was the most frequently affected domain (Figure 1), impaired in 82.1% of patients. Episodic memory and language were each impaired in more than half of the cohort (64.1% and 51.4%, respectively), and global cognitive efficiency was impaired in 43.6%. The least affected domains were praxis/visuoconstruction (33.3%) and visual attention (7.7%).

On domain‐level analysis, the frequency of impairment did not differ significantly between the FCD and FND groups for any cognitive domain. Executive function was the most frequently impaired domain in both groups (14/19 [73.7%] in FCD vs. 18/20 [90.0%] in FND; *p* = 0.235), followed by episodic memory (68.4% vs. 60.0%; *p* = 0.741) and language (47.4% vs. 55.6%; *p* = 0.746). Thus, the pattern of domain‐level impairment did not distinguish FCD from FND. Similarly, no significant between‐group difference was found for any individual test (see Table 6).

**TABLE 6:** Comparison of cognitive‐domain impairment between the FCD and FND groups.

	FCD (*n* = 19)	FND (*n* = 20)	Total (*n* = 39)	*p* value
**Global cognition**	8 (42.1)	9 (45)	17 (43.6)	1.000
**Executive functions**	14 (73.7)	18 (90)	32 (82.1)	0.235
**Visual attention**	1 (5.3)	2 (10)	3 (7.7)	1.000
**Praxis/Visuoconstruction**	7 (36.8)	6 (30)	13 (33.3)	0.741
**Episodicmemory**	13 (68.4)	12 (60)	25 (64.1)	0.741
**Language**	9 (47.4)	10 (55.6)	19 (51.4)	0.746
**Performance validity**	4 (21.1)	1 (5)	5 (12.8)	0.182

A more detailed analysis of the neuropsychological profile in FCD patients will be conducted in a future study, including clustering analyses in a larger sample.

**Illustrative case**

57‐year‐old woman, presenting with a 10‐year history of cognitive complaints. The patient had a history of left‐sided sensorimotor symptoms 15 years earlier that raised suspicion of stroke, although brain imaging was normal and no alternative diagnosis was established. She reported persistent attention and concentration difficulties since then, with stable symptoms over time. Neuropsychological screening during consultation showed marked difficulties, particularly severe naming impairment with semantic errors and poor encoding. Neurological examination revealed left‐sided weakness with positive functional signs, including a positive Hoover sign, consistent with a functional neurological disorder. The presentation was therefore suggestive of a functional cognitive disorder associated with a functional left‐sided sensorimotor deficit.

## 4 Discussion

This study provides a demographic and clinical characterization of patients with FCD with the systematic evaluation of positive FND signs within this population. Notably, we found that 42.1% of patients presenting with a pure cognitive phenotype of FCD showed at least one positive FND sign on neurological examination. Similarly, the pattern of domain‐level cognitive impairment did not distinguish FCD from FND, with comparable rates of impairment across all cognitive domains. These findings are consistent with a rule‐in diagnostic approach and suggest clinical overlap between FCD and FND. They raise the possibility that cognitive symptoms may represent an additional dimension of functional neurological presentations in some patients, alongside motor, sensory, and dissociative seizure phenotypes. However, given the relatively small sample size, these findings should be interpreted cautiously and require replication in larger, controlled studies before firm conclusions can be drawn regarding the nosological relationship between FCD and FND.

While FNDs are defined by the presence of positive clinical signs indicating altered nervous system functioning, these signs are classically described in patients presenting with motor or sensory symptoms; our findings show that they can also be identified in patients with cognitive complaints only. This highlights the importance of actively searching for positive FND signs even when the presenting symptoms are purely cognitive, similarly as previously described in functional dissociative seizures (Cheval et al. 2024).

Until now, the diagnosis of FCD has mainly relied on self‐reported cognitive complaints and evidence of internal inconsistency, such as intact performance at certain times with impaired ability at others. However, such fluctuations can also occur in the early stages of neurodegenerative disorders and may lead to diagnostic uncertainty. Structured assessment of FND‐compatible signs may be useful for describing associated functional neurological features in patients with cognitive symptoms. Such signs are not specific to FCD. For instance, the Hoover's sign has been reported in healthy individuals and in other clinical conditions, such as fatigue, chronic pain, or acute migraine (Coebergh 2025). There should therefore be interpreted within a comprehensive differential diagnostic assessment, particularly when early neurodegenerative disease is a consideration. These signs are simple to assess and can easily be integrated into the standard neurological examination.

The presence of positive FND signs in patients with FCD further supports the conceptual overlap between FCD and other FNDs. It suggests shared vulnerability and potentially common underlying mechanisms between cognitive, motor, sensory, and dissociative FND phenotypes. This overlap also extended to the neuropsychological level, as the pattern of domain‐level cognitive impairment was comparable between FCD and FND. A more detailed characterization of the neuropsychological profile in FCD, including data‐driven clustering analyses in a larger sample, will be addressed in a future study.

Finally, we identified predisposing and precipitating factors that align with the biopsychosocial frame of FNDs. Many patients reported a history of traumatic life events. Mood disorders, such as anxiety and depression, were also frequent comorbidities, in line with findings from larger FCD cohorts, where over half of the patients were clinically depressed (Bhome et al. 2019). Most patients (76%) also reported a triggering factor, most commonly COVID‐19 infection, followed by traumatic brain injury or emotional trauma. These observations raise the possibility that certain triggers may influence the type of functional symptoms expressed. It also raises the possibility that some patients diagnosed with post‐COVID syndrome might actually meet criteria for FCD (Cabreira et al. 2025) (Mavroudis et al. 2025).

### 4.1 Strengths and Limitations

This study includes systematic screening for positive clinical signs of FND with a structured checklist and integrates a full neurological exam into a broad neuropsychological assessment, which gives detailed clinical description of the phenotypes.

Its limitations reside mostly in the small sample size limiting statistical power and subgroup clustering.

On another note, investigators were not blinded to diagnosis or clinical history, increasing the risk of interpretation biases. While such expertise may improve the detection of positive clinical signs, it may also introduce observer expectation bias, particularly in a study specifically examining the relationship between FCD and FND. This may have influenced the frequency with which FND‐compatible signs were identified in our sample. Moreover, we lacked a control group (healthy individuals without cognitive complaints or patients with neurodegenerative disease confirmed by ancillary investigations), which would have allowed a more robust comparison.

Finally, the distinction between FCD and FND is at times somewhat artificial and challenging and largely depends on referral pathways, which allow the findings to be applied to real‐world memory clinic and FND clinic settings.

## 5 Conclusion

This study aims at describing a cohort of patients presenting with cognitive complaints suggestive of FCD. Results reinforce the importance of a comprehensive neurological examination to screen for positive FND signs in FCD. Our findings suggest a possible continuum in the FCD/FND spectrum. Thus, while a dimensional framework may be useful for considering shared clinical and mechanistic features, further research is needed to determine the extent to which FCD and FND reflect common versus distinct underlying processes.

## Author Contributions

**Angeline Maillard**: Writing – review and editing, validation, supervision. **Juliette Palisson**: conceptualization, data curation, writing – review and editing, methodology, investigation, validation. **Olga Goyena**: data curation, formal analysis, writing – original draft, writing – review and editing, investigation, validation. **Inas Al Chare**: writing – review and editing. **Charlotte Joly**: conceptualization, data curation, writing – review and editing, methodology, investigation. **Elsa Mhanna**: writing – review and editing, supervision, data curation, methodology, validation, formal analysis, investigation. **Kenza Benrahmoune**: investigation, data curation. **Paulina Soto**: data curation, formal analysis, writing – original draft, investigation, writing – review and editing, validation. **Beatrice Garcin**: conceptualization, data curation, formal analysis, writing – original draft, methodology, visualization, investigation, supervision, writing – review and editing, project administration, validation, resources.

## Funding

The authors have nothing to report.

## Conflicts of Interest

The authors declare no conflicts of interest.

## Supporting information




**Supplementary Tables**: brb371640‐sup‐0001‐TableS1‐S5.docx

## Data Availability

The data supporting the findings of this study are available from the corresponding author upon request.
