# Supplementary material for: Positive Clinical Signs of Functional Neurological Disorders Are Found in Patients With Functional Cognitive Disorders
Source: Brain Behav. 2026 Aug 19;16(8):e71640. doi: 10.1002/brb3.71640 (PMC13490836; doi:10.1002/brb3.71640)
Supplement: Supplementary file 1 [file BRB3-16-e71640-s001.docx]

**Supplementary material**

Table 1 : Standardized clinical assessment for patients. *Abbreviations: MMSE, Mini-Mental State Examination; MRI, magnetic resonance imaging; WPI, Widespread Pain Index; SSS, Symptom Severity Scale.*

| **Category** | **Assessment items** |
| --- | --- |
| Inclusion criteria | Age 18–80 years; significant cognitive complaint with functional impact; native French speaker; minimum primary education; MMSE >19 |
| Diagnostic classification | Functional cognitive disorder; functional neurological disorder; Alzheimer’s disease; diagnostic status (confirmed/pending) |
| Exclusion criteria | Uncontrolled systemic disease; neurological comorbidity; clinical signs of neurodegenerative disease; MRI abnormalities explaining symptoms |
| Medical history | Relevant medical conditions; current treatments |
| Psychiatric history | Depression; anxiety; psychological or physical trauma; other psychiatric disorders |
| Neurological history | Personal and family neurological or cognitive history |
| Sociodemographic data | Education/profession; marital status; children; tobacco and alcohol use |
| Symptom onset | Date of onset; primary symptom domain |
| Cognitive complaints | Memory; attention; language; executive functions; visuospatial abilities; praxis; orientation; behavioral changes |
| Non-cognitive symptoms | Functional seizures; movement disorders; sensory symptoms; pain; fatigue; balance; speech or swallowing difficulties |
| Neurological examination | Pyramidal, cerebellar, parkinsonian signs; visual impairment |
| Functional neurological signs | Gait abnormalities; limb inconsistency; Hoover/abductor signs; distractibility; entrainment; fixed dystonia |
| Additional measures | Fibromyalgia indices (WPI, SSS), when available |

Table 2 : Full standardized clinical assessment for patients. *(filled during assessment)*

- SOCIODEMOGRAPHIC DATA

| Patient ID |  |
| --- | --- |
| Group | - Functional Neurological Disorder - Functional Cognitive Disorder |
| Gender | - Women - Man - Other |
| Date of birth |  |
| Age ≥ 18 and ≤ 80 years | - Yes - No |
| Mother language | - French - Other: |
| Educational level CEP or higher | - Yes - No |
| Number of years of schooling |  |
| Profession |  |
| Marital status | - Single - Married - Divorced - Widowed |
| Nº of children |  |

- CLINICAL VARIABLES

| Date of interview |  |
| --- | --- |
| Date of neuropsychological assessment |  |
| Date of symptom onset |  |
| Date of onset of non-cognitive complaints (if not simultaneous) |  |
| Main complaint | - Cognitive - Motor - Sensory - Pain or fatigue |
| MRI findings | - Normal - Abnormal - Not performed |
| Brain PET scan | - Normal - Abnormal - Not performed |
| Lumbar puncture | - Normal - Abnormal - Not performed |
| EMG | - Normal - Abnormal - Not performed |
| EEG | - Normal - Abnormal - Not performed |
| Somatosensory evoked potentials | - Normal - Abnormal - Not performed |

- MEDICAL HISTORY

| Substance use | - Tobacco use - Alcohol use - Other substances |
| --- | --- |
| Relevant Medical History |  |
| Neurological History | - Headache - Epilepsy - Others: |
| Psychiatric History | - Depression - Anxiety - Psychosis - Physical or psychological trauma - Sleep disorders - Other |
| History of autoinmune disease | - Yes: ______________ - No |
| Family history of neurological diseases | - Yes: ______________ - No |
| Current medical treatment |  |
| Any medical treatment affecting cognition | - Yes: ______________ - No |
| A precipitating factor identified | - No - Infection: _______________ - Accident: _______________ - Work-related: ____________ - Other: _________________ |

- SYMPTOMS: Check if present
  - Dissociative functional seizures
  - Dystonia
  - Tremor
  - Motor déficit
  - Sensory symptoms
  - Pain
  - Fatigue
  - Dizziness
  - Speech or phonation disorder
  - Dysphagia
  - Urinary symptoms
  - Digestive symptoms
- COGNITIVE COMPLAINTS: Check if present
  - Memory
  - Attention
  - Brain fog
  - Language
  - Organization
  - Orientation
  - Visual disturbances
  - Praxies
  - Behavior problems
  - Recognition of people
- NEUROLOGICAL EXAMINATION

| Rigidity-akinetic syndrome | - Yes - No |
| --- | --- |
| Cerebellar syndrome | - Yes - No |
| Pyramidal syndrome | - Yes - No |
| Positive clinical sign of FND | - Yes - No |
| Positive clinical sign of FND: Check if present | Walking   - Sudden knee buckling - Dragging monoplegic leg - Non-economic posture - Hesitant/cautios gait - Fall toward support   Motor   - Excessive slowness without bradykinesia - Collapsing/give-away weakness - Drift without pronation - Hoover’s sign - Abductor sign - Cocontraction of antagonist muscle - Spinal injury test - Chair test - Abductor finger sign - Superior limb flexion/extension sign (Hoover equivalent)   Sensory   - Midline splitting - Splitting of vibration - Systematic failure during sensory testing   Others   - Distractibility - Entrainment effect - Oculomotor complaint/eye movement abnormalities during examination |

Table 3: How-To: The check list of positive clinical signs of FND (based on Aybeck et al 2022 (9) and Daum et al 2014 (10))

|  | **How to test ?** |
| --- | --- |
| **Walking** | |
| Hesitant or cautious walking | Look for disproportionate hesitation and caution in gait (contrasting with good balance, strength, sensation) |
| Non-economic posture | Look for postures during gait that require good balance and strength such as flexed knees |
| Sudden knee buckling | Look for sudden buckling of the knee, usually with each step. Extreme cases will show knee touching the floor at each step |
| Fall towards support | The patient tends to fall in the direction of support (wall, furniture) |
| Dragging monoplegic leg | The weak leg is “dragged” like a piece of wood/ inanimate object, without spastic circumduction, usually along the floor surface |
| **Motor** | |
| Collapsing/ Give-away weakness | When testing strength against resistance; initially good and then sudden loss of resistance from the patient |
| Co-contraction of antagonist muscles | When testing strength, no movement at the joint (elbow, for example) occurs because co-contraction of agonist and antagonist is observed |
| Spinal injury test | Passively put both legs in a flexed position, sole of feet touching the bed during a lying position; observe if the weak leg stays in this position (functional) or falls back on the bed (“organic” |
| Chair test | In case of severe gait disorder, ask the patient to propel a chair with wheels; movements of the legs will be better than during gait |
| Excessive slowness | Look for disproportionate slowness in gait (slow stepping movements contrasting with lack of limb bradykinesia) |
| Hoover sign | Ask to flex the healthy hip against resistance and observe/feel the strength of hip extension of the weak leg (if patient lying: examiner’s hand under the heel, if sitting under the thigh). Compare with voluntary hip extension of the weak leg: if involuntary strength >voluntary strength, Hoover is positive |
| Abductor sign | Ask to abduct both legs against resistance: observe/ feel if involuntary abduction of the weak leg occurs (functional) or not (“organic”) |
| Abductor finger sign | In severe unilateral hand weakness, ask the patient to abduct the fingers of the healthy hand against resistance; observe if involuntary abduction of the 5th finger in the weak hand occurs (functional) or not (“organic”) |
| Drift without pronation | Arms stretched out, palms up in a full supination position, fingers adducted, eyes closed for 10 s: if a downward drift is seen, observe if a movement of pronation also occurs |
| Superior limb flexion/extension sign (Hoover equivalent) | Arms flexed at 30°, forearms held near the wrists by examiner. Ask patient to flex the healthy arm against resistance and observe/feel if increased extension of the weak arm occurs (functional) or not (“organic”). Then ask patient to flex weak arm and observe if extension of healthy arm occurs (“organic”) or not (functional) |
| Tremor with functional characteristic below | |
| Distractibility | Pause during ballistic movement or during other motor/mental task or change in amplitude and frequency |
| Entrainment effect | Ask to imitate tapping motion with one hand and observe the change in tremor frequency on the other |
| “Whack a mole” sign | Immobilize the limb affected by tremor : the tremor appears in another body segment (head, trunk, other arm, or legs). |
| Frequency fluctuations | Observe changes in tremor frequency during history taking/ examination/arriving or leaving the examination room: periods of unexplained improvement/ disappearance of symptom |
| **Sensitive** | |
| Systematic failure | Patients always fails in a discriminative task (eg, pin or prick/cold-hot/upgoing or downgoing joint) |
| Midline splitting | Touch the trunk : the patient report exact splitting of sensation in the midline |
| Spliting of vibration | Place the tuning fork on a flat bone (sternum or frontal bone): patient report a difference in the sensation of the vibration over the left compared to the right side |
| **Other** | |
| Oculomotor complaint / Eye movement abnormalities during examination | Do a classical oculomotor exam : excessive blinking, effortful facial expression, increased latency, gaze deviation, limited range, absent frontalis contraction during upgaze or complaint about the difficulty of performing the requested test |
| Fixed dystonia | New onset dystonic posture without possibility to move the joint of the affected limb |

Table 4: Neuropsychological assessment battery

| **Cognitive domain** | **Assessment tools** |
| --- | --- |
| Global cognition | Mini-Mental State Examination (MMSE) |
| Executive functions | Verbal and visual working memory tasks (Mémoire des chiffres WAIS-IV, mémoire spatiale MEM-III); Batterie Rapide d’Efficience Frontale (BREF); Trail Making Test (A and B); Stroop test (GREFEX and/or Victoria); SimiCAT; verbal fluency (; phonemic: letter P) |
| Episodic memory | Verbal and Visual Memory Test (TMV); FCSRT, Rey–Osterrieth Complex Figure (delayed recall); DMS-48; Rey 15-Item Test |
| Language | 40-item Naming Test (DO-40) ; verbal fluency (semantic animals) |
| Attention and visuospatial functions | Bells Cancellation Test |
| Praxis | Ideomotor and constructive praxis tasks (Batterie des Praxies de Mahieux, Rey–Osterrieth Complex Figure (copy), Figures de la BEC-96) |
| Self-report measures | Childhood Trauma Questionnaire (CTQ); Hospital Anxiety and Depression Scale (HADS) |

Table 5: Past Medical History and Comorbidities

| **Past medical history** | **FCD (n = 19)** | **FND (n = 20)** | **Total (n = 39)** |
| --- | --- | --- | --- |
| **Neurological disorders** | 8 | 8 | 16 |
| Headache | 3 | 4 | 7 |
| Neurovascular disease | 3 | 0 | 3 |
| Infectious* | 0 | 2 | 2 |
| Epilepsy | 1 | 1 | 2 |
| Other neurological disorders** | 1 | 4 | 5 |
| **Immune-mediated disorders** | 5 | 4 | 9 |
| Hypothyroidism | 0 | 3 | 3 |
| Type 1 diabetes mellitus | 2 | 0 | 2 |
| Other immune-mediated disorders*** | 3 | 2 | 5 |
| **Other functional somatic symptoms** | 4 | 6 | 10 |
| Fibromyalgia | 3 | 4 | 7 |
| Irritable bowel syndrome | 1 | 2 | 3 |
| Chronic fatigue syndrome | 2 | 0 | 2 |
| **Psychiatric disorders** | 15 | 12 | 27 |
| Depression | 15 | 9 | 24 |
| Anxiety | 8 | 9 | 17 |
| Psychotic disorder | 1 | 0 | 1 |
| Physical or psychological trauma | 11 | 11 | 22 |
| **Other medical conditions** | 18 | 18 | 36 |
| Metabolic conditions | 6 | 3 | 9 |
| Cardiovascular disease | 5 | 4 | 9 |
| Surgical history | 5 | 6 | 11 |
| Insomnia | 7 | 12 | 19 |
